# Initial therapeutic results of atezolizumab plus bevacizumab for unresectable advanced hepatocellular carcinoma and the importance of hepatic functional reserve

**DOI:** 10.1002/cam4.5145

**Published:** 2022-08-14

**Authors:** Tetsu Tomonari, Joji Tani, Yasushi Sato, Hironori Tanaka, Takahiro Tanaka, Tatsuya Taniguchi, Morishita Asahiro, Koichi Okamoto, Masahiro Sogabe, Hiroshi Miyamoto, Naoki Muguruma, Tsutomu Masaki, Tetsuji Takayama

**Affiliations:** ^1^ Department of Gastroenterology and Oncology Tokushima University Graduate School of Biomedical Sciences Tokushima Japan; ^2^ Department of Gastroenterology and Neurology Kagawa University Graduate School of Medicine Kagawa Japan; ^3^ Department of Community Medicine for Gastroenterology and Oncology Tokushima University Graduate School of Biomedical Sciences Tokushima Japan

**Keywords:** atezolizumab, bevacizumab, hepatocellular carcinoma

## Abstract

**Aim:**

We analyzed the association between the modified albumin–bilirubin (mALBI) grade and therapeutic efficacy of atezolizumab plus bevacizumab (Atezo+Bev) for the treatment of unresectable hepatocellular carcinoma (u‐HCC).

**Methods:**

In this retrospective observational study, we included 71 u‐HCC patients treated with Atezo+Bev between September 2020 and September 2021. Patients were grouped corresponding to the mALBI grade at the start of treatment (mALBI 1+2a or mALBI 2b+3) and analyzed for therapeutic effect and the transition rate to secondary treatment.

**Results:**

According to the Response Evaluation Criteria in Solid Tumors, the overall response rate was significantly higher for the mALBI 1+2a group, than for the mALBI 2b+3 group, with 26.2% and 3.4%, respectively. The progression‐free survival (PFS) was significantly longer in the mALBI 1+2a group (10.5 months) than in the mALBI 2b+3 group (3.0 months). In the multivariate analysis, an mALBI of 1+2a was found to be an independent factor of PFS. The rate of second‐line treatment with multi‐targeted agents was also significantly higher in the mALBI 1+2a group.

**Conclusions:**

In real‐world practice, Atezo+Bev treatment might have higher therapeutic efficacy in u‐HCC patients with mALBI 1+2a.

## Graphical abstract

Our data suggest that Atezo+Bev treatment has a good anti‐tumor effect on mALBI 1+2a and a reasonable transition rate to the subsequent molecular‐targeted agents treatment.

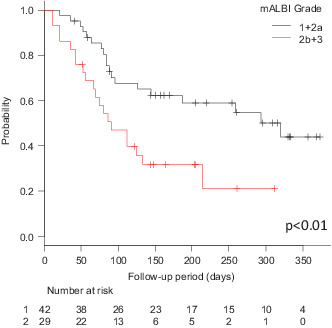

## 1 INTRODUCTION

Hepatocellular carcinoma (HCC) is the most common histological form of primary liver cancer with a major cause of cancer‐related death globally.^1^, ^2^ Over recent years, substantial treatment advancements have been achieved in drug therapy by introducing molecular‐targeted agents (MTA) as well as immune checkpoint inhibitors (ICIs) for unresectable advanced hepatocellular carcinoma (u‐HCC). A global, randomized phase III trial, IMbrave 150 study, aimed to evaluate the efficacy of the atezolizumab plus bevacizumab (Atezo+Bev) compared with sorafenib alone as a first‐line treatment for u‐HCC.^3^ The trial showed significant survival benefits (progression‐free survival [PFS], hazard ratio (HR), 0.59; 95% CI, 0.47–0.76; overall survival (OS), HR, 0.58; 95% CI, 0.42–0.79) in patients treated with Atezo+Bev. With these positive results, the Atezo+Bev regimen is established as a first‐line treatment for u‐HCC.^4^, ^5^

The Child–Pugh score system is the most common global index for the evaluation of the hepatic functional reserve. Recently, the albumin–bilirubin (ALBI) grading system has been used as another to the Child–Pugh score to assess liver function in HCC patients.^6^ More recently, various studies have reported that modified ALBI (mALBI) grades are useful in evaluating hepatic functional reserve in patients with u‐HCC and are associated with the therapeutic effect of drug therapy on u‐HCC.^7^, ^8^, ^9^, ^10^

To date, the efficacy and safety of Atezo+Bev as first‐ or later‐line therapy in actual clinical practice and the impact of liver function reserve on these outcomes have not yet been fully investigated. Moreover, there have been no reports to evaluate the relationship between the therapeutic effect of Atezo+Bev and mALBI grade. In this study, we investigated the correlation between the treatment effect of Atezo+Bev and mALBI grade in u‐HCC patients treated with Atezo+Bev in the clinical setting.

## 2 METHODS

### 2.1 Selection criteria and diagnostic criteria for hepatocellular carcinoma

We retrospectively evaluated the efficacy and safety of Atezo+Bev (Chugai Pharmaceutical Co. Ltd) therapy for u‐HCC conducted at Tokushima University Hospital and Kagawa University Hospital from September 2020 to September 2021.

The Selection criteria are based on the IMbrave150 study.^3^ The eligible patients had evaluable nodules by the Response Evaluation Criteria in Solid Tumors (RECIST)^11^ and modified RECIST (mRECIST) criteria,^12^ with Child‐Pugh (CP) class A, the Eastern Cooperative Oncology Group Performance Status (ECOG‐PS) score of 0 or 1^13^ and Barcelona Clinic Liver Cancer (BCLC) stage B or C.^14^

In patients with CP class B, Atezo+Bev was administered according to the criteria of the GO30140 study.^15^ The diagnosis of HCC was made according to the guidelines of the Liver Cancer Study Group in Japan.^16^ The diagnosis of HCC was confirmed on the basis of histological or characteristic radiological findings, such as washout patterns in images of the arterial enhancement and equilibrium phases typical of tumors by imaging modalities, such as gadolinium‐enhanced magnetic resonance imaging or dynamic computed tomography. Treatment selection after the radiological PD of the Atezo+Bev therapy, in the case of CP‐A and PS‐0,1 selected MTAs according to each clinical trial,^17^, ^18^, ^19^, ^20^ if it did not meet the criteria, TAE/TACE and hepatic arterial infusion chemotherapy (HAIC) were selected. Best supportive care (BSC) was selected when it was judged that tolerability to TAE/TACE and HAIC was poor or based on the needs of patients.

This study was conducted in accordance with the guidelines of the 1975 Declaration of Helsinki. The research protocol for this study was approved by the Institutional Ethics Committee of the University of Tokushima Hospital (number: 3816) and the participating institution.

### 2.2 Treatment protocol

For the Atezo+Bev therapy, the patients were administrated intravenous 1200 mg atezolizumab and 15 mg/kg bevacizumab every 3 weeks. When serious adverse events (AEs), i.e., unacceptable grade 2 AEs or grade 3 AEs, were observed, Atezo + Bev therapy was discontinued until the patient improved to a milder Grade compared to the onset of AEs.

### 2.3 Patient outcomes and assessment

Patients included in the analysis were those who had received at least 6 weeks of Atezo+Bev therapy. Safety was evaluated by reviewing hematological and biochemical findings by blood tests and urinalysis and physical findings. Drug‐induced adverse events were evaluated using the Common Terminology Criteria for Adverse Events version 5.0. The response rate to Atezo+Bev treatment was assessed by RECIST and mRECIST every 6 weeks. Overall response rate (ORR) was defined as complete response (CR)+partial response (PR), and disease control rate (DCR) was determined as CR+PR+stable disease (SD). PFS was defined as the time from the first day of treatment with Atezo+Bev to the date of radiological progression or death from various causes.

### 2.4 Hepatic functional reserve

Hepatic functional reserve was evaluated using Child‐Pugh scoring and mALBI grading. The mALBI grade was calculated using total bilirubin and serum albumin levels.^6^ Patients who withdrew or discontinued Atezo+Bev by 9 weeks were excluded from the analysis of the change in ALBI score.

### 2.5 Statistical analysis

Binomial variables were tested by Fisher's exact test, and continuous variables were examined by Mann–Whitney *U* test. Statistical significance was set at *p* value <0.05. PFS was analyzed by Kaplan–Meier method and log‐rank test. All statistical analyses were conducted using Easy R version 1.^21^ Multivariate analysis was performed using variables reported to influence u‐HCC treatment.^22^, ^23^, ^24^ The COX proportional hazards model was used for multivariate analysis. Furthermore, multivariate analysis included factor, which was *p* < 0.1 in univariate analysis.

## 3 RESULTS

### 3.1 Patient characteristics

Seventy‐five patients were treated with Atezo+Bev between September 2020 and September 2021. Four patients were excluded, because they did not complete the initial radiological evaluation; therefore, 71 were examined in this study. Table 1 summarizes the baseline characteristics of the study population. The median age of the patients was 71 years (Quartile, 66–79 years), and 13 (18.3%) were female. Of all patients, 8 (11.3%) were HBV antigen positive, and 30 (42.3%) were HCV antibody positive; ECOG‐PS was 0 in 42 (59.2%) patients. Furthermore, the median alpha‐fetoprotein (AFP) level was 178 ng/ml (quartiles, 11–1243 ng/ml) and the pre‐treatment Child‐Pugh score was 5 in 38 patients, 6 in 27 patients, 7 in 3 patients, and 8 in 3 patients. Of the 71 patients, 34 were MTA‐naive (first‐line treatment), 15 were second‐line treatment, 10 were third‐line treatment, 8 were fourth‐line treatment, and 4 were fifth‐line treatment. Furthermore, the mALBI grade at the start of Atezo+Bev therapy was 1 point in 18 patients, 2a in 24 patients, 2b in 27 patients, and 3 in 2 patients, and the BCLC stage was stage A in 4 patients, stage B in 24 patients, and stage C in 43 patients. When the characteristics of these 71 patients at baseline were compared between mALBI grade 1+2a (*N* = 42) and 2b+3 (*N* = 29), there was no significant difference in patient characteristics and other variables between the two groups (Table 1).

**TABLE 1:** Characteristics of patients with unresectable advanced hepatocellular carcinoma treated with atezolizumab plus bevacizumab therapy

Characteristics	All (*n* = 71)	mALBI 1+2a (*n* = 42)	mALBI 2b+3 (*n* = 29)	*p*‐value
Observation period, median [quartiles], (days)	202 [109–265]	194 [141–303]	187 [102–258]	0.51
Age, median [quartiles], (years)	71 [66–79]	72 [67–78]	79 [67–80]	0.21
Sex (male/female), *n*	58/13	34/8	24/5	1
ECOG‐PS (0/1), *n*	42/29	26/16	19/10	0.09
Etiology (HBV/HCV/NBNC), n	8/30/33	7/20/15	1/10/18	0.20
Platelets, median [quartiles], (10^4^/μl)	15.2 [10.6–18.7]	16.4 [13.5–19.4]	11.3 [9.5–17.2]	0.09
M2BpGi [quartiles] (C.O.I.)	2.22 [1.06–4.49]	1.95 [0.84–2.75]	2.80 [1.60–5.74]	0.07
Child–Pugh score (5/6/7/8), *n*	38/27/3/3	34/8/0/0	4/19/3/3	
mALBI Grade (1/2a/2b/3), *n*	18/24/27/2	18/24/0/0	0/0/27/2	
Intrahepatic nodules (none/1/2–7/>7)	8/13/27/23	7/16/6/13	1/11/7/10	0.3
Maximum diameter of intrahepatic nodule (none/≤50/>50) (mm)	5/40/26	0/19/7	0/14/5	0.32
Portal vein invasion (absent/present), *n*	56/15	35/7	21/8	0.38
Extrahepatic spread (absent/present), *n*	47/24	16/26	8/21	0.48
AFP, median [quartiles] (ng/ml)	178 [11–1243]	79 [7.5–1006]	368 [79–1459]	0.13
BCLC stage (A/B/C), *n*	4/24/43	2/17/23	2/7/20	0.37
Treatment line (first line/second line/third line/fourth line fifth line), *n*	34/15/10/8/4	19/8/6/6/3	15/7/4/2/1	0.83
Previously used drugs (sorafenib/regorafenib/lenvatinib ramucirumab)	(22/14/36/9)	(16/10/22/5)	(6/4/14/4)	–

Abbreviations: AFP, alpha‐fetoprotein; BCLC, Barcelona Clinic Liver Cancer; ECOG‐PS, Eastern Cooperative Oncology Group performance status; HBV, hepatitis B virus; HCV, hepatitis C virus; M2BPGi, mac‐2 binding protein glycosylation isomer; mALBI, modified albumin–bilirubin; NBNC, non‐B non C.

### 3.2 Treatment effect

The median observation period of this study was 202 (50–370) days. The results of treatment response by RECIST (ver 1.1), and mRECIST criteria are shown in Table 2. Seventy‐one patients had measurable nodules assessable on enhanced CT/MRI 6 weeks after initiation of Atezo+Bev therapy. The RECISTver1.1 evaluation showed that 12 (16.9%) of the 71 patients presented with PR, 42 (59.2%) with SD, and 17 (23.9%) with PD. The overall ORR and DCR were 16.9% and 76.1%, respectively. When examining the ORR by mALBI group, the mALBI 1+2a group (26.2%) was significantly better than the mALBI 2b+3 group (3.4%) (*p* = 0.02). On the other hand, DCR was similar in the mALBI 1+2a (78.8%) and mALBI 2b+3 (72.4%) groups.

**TABLE 2:** Response to treatment with Atezolizumab plus bevacizumab for hepatocellular carcinoma

Evaluation (RECISTver1.1)	All *n* (%) (*n* = 71)	mALBI 1+2a *n* (%) (*n* = 42)	mALBI 2b+3 *n* (%) (*n = 29*)	Evaluation (mRECIST)	All *n* (%) (*n* = 71)	mALBI 1+2a *n* (%) (*n = 4*2)	mALBI 2b+3 *n* (%) (*n = 29*)
Complete response	0 (0)	0 (0)	0 (0)	Complete response	2 (2.8)	1 (2.4)	1 (3.4)
Partial response	12 (16.9)	11 (26.2)	1 (3.4)	Partial response	14 (19.7)	11 (26.2)	3 (10.4)
Stable disease	42 (59.2)	22 (52.4)	20 (69.0)	Stable disease	39 (54.9)	21 (50.0)	18 (62.1)
Progressive disease	17 (23.9)	9 (21.4)	8 (27.6)	Progressive disease	16 (22.5)	9 (21.4)	7 (24.1)
Objective response rate (%)	16.9	26.2*	3.4	Objective response rate (%)	22.5	28.6	13.8
Disease control rate (%)	76.1	78.8	72.4	Disease control rate (%)	77.5	78.8	75.9

Abbreviations: mALBI, modified albumin–bilirubin; mRECIST, modified Response Evaluation Criteria in Solid Tumors; RECIST, Response Evaluation Criteria in Solid Tumors.

* *p* < 0.05 versus mALBI 2b+3.

According to mRECIST, CR and PR were present in 2 (2.8%) and 14 (19.7%) patients, respectively (ORR: 22.5%). SD was observed in 39 patients (54.9%) and PD in 16 patients (22.5%) (DCR: 77.5%). When examining the results by mALBI group, the ORR was 28.6% in the mALBI 1+2a group and 13.8% in the mALBI 2b+3 group, which was not significantly different (*p* = 0.16). DCR was similar in the mALBI 1+2a (78.8%) and mALBI 2b+3 (75.9%) groups.

**FIGURE 1:**
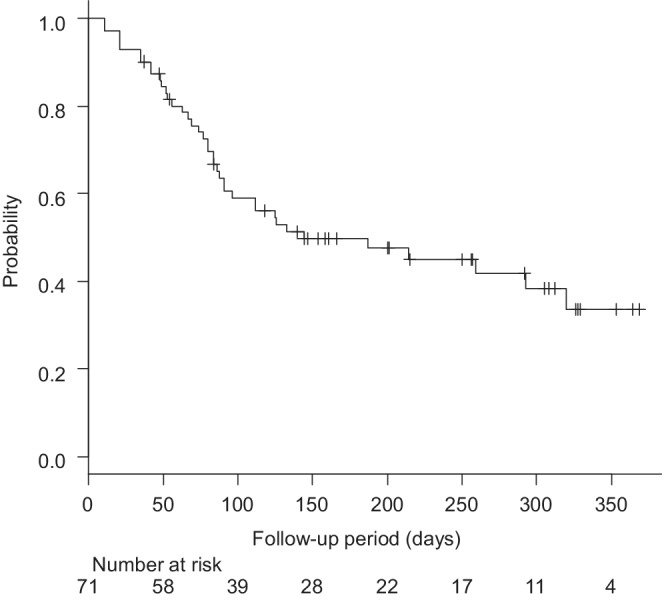
The progression‐free survival of all 71 patients treated with atezolizumab + bevacizumab analyzed using Kaplan–Meier curve.

**FIGURE 2:**
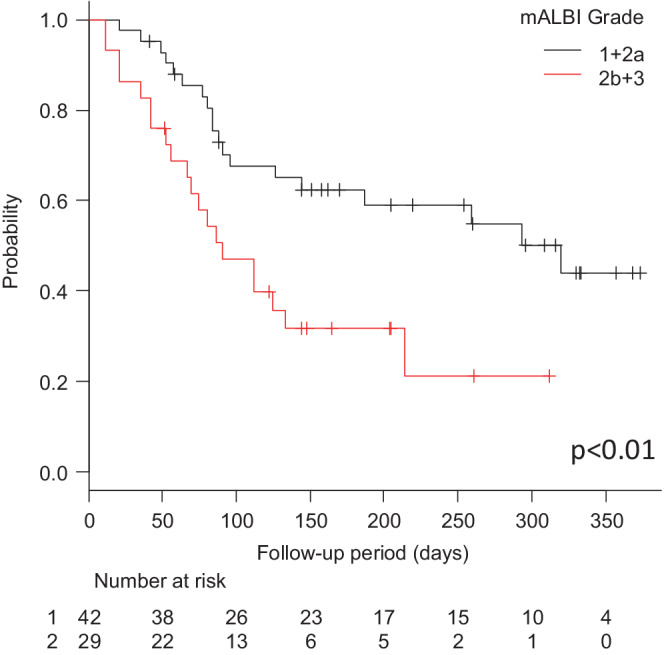
Kaplan–Meier curves for progression‐free survival in atezolizumab plus bevacizumab‐treated patients stratified by mALBI Grade. There was a statistically significant difference between the mALBI 1+2a group and the mALBI 2b+3 group.

The median PFS according to the RECIST was 144 days (4.7 months) (Figure 1). The median PFS was significantly longer in the mALBI 1+2a group (320 days [10.5 months], 95% CI 126 ‐not applicable [NA] days) versus the mALBI 2b+3 group (91 days [3.0 months], 95% CI 56–133 days, HR 2.086; 95% CI 1.054–4.130) (*p* < 0.01) (Figure 2). The median PFS was not significantly different between the first‐line group (NA days, 95% CI 85‐NA days) and the late‐line group (134 days [4.4 months], 95% CI 85–292 days, HR 0.761; 95% CI 0.398–1.451) (Figure S1) (*p* = 0.41). The median PFS according to the mRECIST was 154 days (5.1 months) (Figure S2). Although 11 patients died within the observation period, the median overall survival was not available for analysis.

### 3.3 Number of treatments and duration of treatment

The analysis of the mean number of doses and duration of Atezo+Bev (*n* = 71) showed that the mean number of medications of Atezo was 5.0 times and the duration of medications was 108 days. The average number of Bev medications was 4.8, and the duration was 105 days. Furthermore, when the number of Atezo+Bev medications and the duration of medications in the mALBI 1+2a (*n* = 42) and 2b+3 groups (*n* = 29) were analyzed, the number of Atezo medications and the duration of medications in the mALBI 1+2a group were 5.5 times and 119.4 days, and the number of Bev medications was 5.3 times and 116 days, while in the 2b+3 group, the number of Atezo+Bev medications In both the Atezo and Bev studies, the mALBI 1+2a group had a higher number of doses and a longer duration of dosing. Next, when analyzing the association between the best anti‐tumor effect by RECIST and the number of medications and duration of Atezo+Bev, we found that in the PR group (*n* = 12), Atezo was administered seven times and the duration of medication was 153.2 days, and Bev was administered 6.6 times and 145 days, while in the SD group (*n* = 42), Atezo was administered 5.4 times and the duration of medication was 117.2 days, Bev was administered 5.2 times and the duration of medication was 113.5 days. In the PD group (*n* = 17), Atezo was administered twice and the duration was 42.4 days, and Bev was administered twice and the duration was 42.4 days, indicating that the patients with better anti‐tumor efficacy received more medication and the duration was longer. (Table 3).

**TABLE 3:** Duration and number of treatments in Atezolizumab plus bevacizumab

Evaluation (average)	All (*n* = 71)	mALBI 1+2a (*n* = 42)	mALBI 2b+3 (*n* = 29)	Evaluation (average)	PR (*n* = 12)	SD (*n* = 42)	PD (*n* = 17)
Atezolizumab				Atezolizumab			
Median duration of medication, days (range)	108 (21–336)	119.4 (21–336)	86 (21–273)	Median duration of medication, days (range)	153.2 (21–336)	117.2 (21–308)	42.4 (21–91)
Treatment time (mean times)	5	5.5	3.8	Treatment time (mean times)	7	5.4	2
Bevacizumab				Bevacizumab			
Median duration of medication, days (range)	105 (21–336)	116 (21–336)	82 (21–273)	Median duration of medication, days (range)	145 (21–336)	113.5 (21–308)	42.4 (21–91)
Treatment time (mean times)	4.8	5.3	3.8	Treatment time (mean times)	6.6	5.2	2

### 3.4 Treatments and transition rate following the progression of Atezo+Bev

During the observation period, PD was radiologically determined in 41 of 71 patients treated with Atezo+Bev (mALBI 1+2a, *n* = 25; mALBI 2b, *n* = 16) (Table 4). Nineteen patients (46.3%) received post‐treatment with MTA (lenvatinib [LEN], *n* = 8; cabozantinib [CAB], *n* = 6; ramucirumab [RAM], *n* = 4; regorafenib [REG], *n* = 1), followed by BSC (*n* = 13; 31.7%), transarterial embolization or chemoembolization (TAE/TACE, *n* = 8; 19.5%), and HAIC (*n* = 1; 2.4%). When examined by mALBI group, 15 patients (60.0%) in the mALBI 1+2a group were later treated with MTA (LEN *n* = 6; CAB *n* = 5; RAM *n* = 3; REG *n* = 1), followed by BSC (*n* = 6; 24%), TAE/TACE (*n* = 3; 16%), and HAIC (*n* = 1; 4%) (*n* = 1, 4%). In the mALBI 2b+3 group, four patients (25.0%) were later treated with MTAs (LEN, *n* = 2; CAB, *n* = 1; RAM, *n* = 1), followed by BSC (*n* = 7, 43.8%) and TAE/TACE (*n* = 5, 31.2%). The rate of transition to MTAs treatment was significantly better in mALBI 1+2a (60%) than in mALBI 2b (25.0%) (*p* = 0.02). The change in hepatic reserve function at the end of treatment by liver reserve was analyzed using the mALBI score. mALBI score at the beginning was −2.549 in the mALBI 1+2a group and −2.430 at the point of imaging PD, (*p* = 0.08) which was not statistically significant, while in the mALBI 2b group mALBI at the beginning of treatment score was −1.961 and −1.486 at the point of imaging PD, a statistically significant difference (*p* < 0.01).

**TABLE 4:** Post treatment after imaging progression on atezolizumab plus bevacizumab therapy

Treatment	All, *n* (%) (*n* = 41)	mALBI 1+2a, *n* (%) (*n* = 25)	mALBI 2b, *n* (%) (*n* = 16)	*p*‐value
MTAs	19 (46.3)	15 (60.0)	4 (25.0)	0.02
TAE/TACE	8 (19.5)	3 (12.0)	5 (31.2)	0.26
HAIC	1 (2.4)	1 (4.0)	0 (0)	1
BSC	13 (31.7)	6 (24.0)	7 (43.8)	0.36

Abbreviations: BSC, best supportive care; HAIC, hepatic arterial infusion chemotherapy; mALBI, modified albumin–bilirubin; MTAs, multi‐targeted agents; TAE/TACE, transcatheter embolization/chemoembolization.

### 3.5 Adverse events

AEs observed during treatment with Atezo+Bev are shown in Table 4. The most frequent AE was decreased appetite (all grades, *n* = 23/71 [32.4%], grade 3, *n* = 1/71 [1.4%]), followed by fatigue (all grades, *n* = 19/71 [26.8%], grade 3, *n* = 4/71 [5.6%]), proteinuria (all grades, *n* = 15/71 [21.1%], grade 3, *n* = 5 [7.0%]), hypertension (all grades, *n* = 9/71 [12.7%], grade 3, *n* = 1 [1.4%]), and fever (all grades, *n* = 8/71 [11.3%], grade 3, *n* = 0 [0%]).

During the Atezo+Bev treatment observation period, 23 patients (32.4%, 23/71) were withdrawn or discontinued due to AEs. The frequency of AEs resulting in dose withdrawal or discontinuation was significantly higher in the mALBI 2b+3 group (51.7%, 15/29 patients) than in the mALBI 1+2a group (19.0%, 8/42 patients) (*p* < 0.01) (Table 5).

**TABLE 5:** Adverse events associated with atezolizumab plus bevacizumab treatment

Event	All (*n* = 71)		mALBI 1+2a *(n* = 42)		mALBI 2b+3 (*n* = 29)		*p*‐value	
	Any grade, *n* (%)	Grade 3, *n* (%)	Any grade, *n* (%)	Grade 3, *n* (%)	Any grade, *n* (%)	Grade 3, *n* (%)	Any grade, *n* (%)	Grade 3, *n* (%)
Decreased appetite	23 (32.4)	1 (1.4)	10 (23.8)	0 (0)	13 (44.8)	1 (3.4)	0.08	0.41
Fatigue	19 (26.8)	4 (5.6)	10 (23.8)	0 (0)	9 (31.0)	4 (13.8)	0.59	0.13
Proteinuria	15 (21.1)	5 (7.0)	10 (23.8)	3 (7.1)	5 (17.2)	2 (6.9)	0.57	0.59
Hypertension	9 (12.7)	1 (1.4)	7 (16.7)	0 (0)	2 (6.9)	1 (3.4)	0.32	0.41
Fever	8 (11.3)	0 (0)	7 (16.7)	0 (0)	1 (3.4)	0 (0)	0.18	–
Increased transaminase	7 (9.9)	0 (0)	3 (7.1)	1 (2.4)	4 (13.8)	0 (0)	0.43	–
Rash	6 (8.5)	1 (1.4)	5 (12.0)	1 (2.4)	1 (3.4)	0 (0)	0.40	–
Diarrhea	3 (4.2)	1 (1.4)	2 (4.8)	1 (2.4)	1 (3.4)	0 (0)	0.26	–
Adrenal insufficiency	2 (2.8)	0 (0)	2 (4.8)	0 (0)	0 (0)	0 (0)	0.51	–
Interstitial pneumonia	2 (2.8)	1 (1.4)	2 (4.8)	1 (2.4)	0 (0)	0 (0)	0.51	–
Hypothyroidism	2 (2.8)	0 (0)	1 (2.4)	0 (0)	1 (3.4)	0 (0)	0.72	–
Increased blood bilirubin	1 (1.4)	1 (1.4)	1 (2.4)	1 (2.4)	0 (0)	0 (0)	0.16	–
Decreased platelet count	1 (1.4)	0 (0)	1 (2.4)	0 (0)	0 (0)	0 (0)	0.57	–
Gastrointestinal bleeding	1 (1.4)	0 (0)	1 (2.4)	0 (0)	0 (0)	0 (0)	–	–
AE related dose withdrawal or discontinuation	23 (32.4)	–	8 (19.0)	–	15 (51.7)	–	0.01	

### 3.6 Effect of Atezo+Bev on ALBI score over the treatment period

**FIGURE 3:**
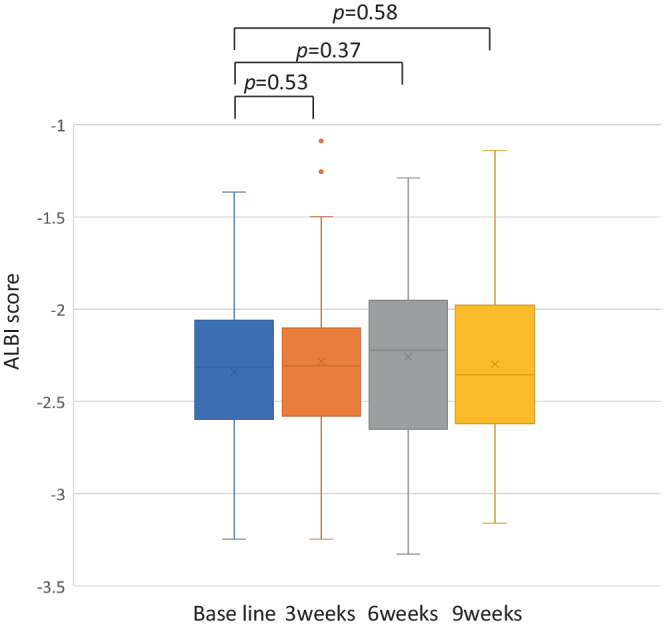
The transition of hepatic functional reserve after the administration of treatment with atezolizumab+bevacizumab at baseline, 3, 6, and 9 weeks.

**FIGURE 4:**
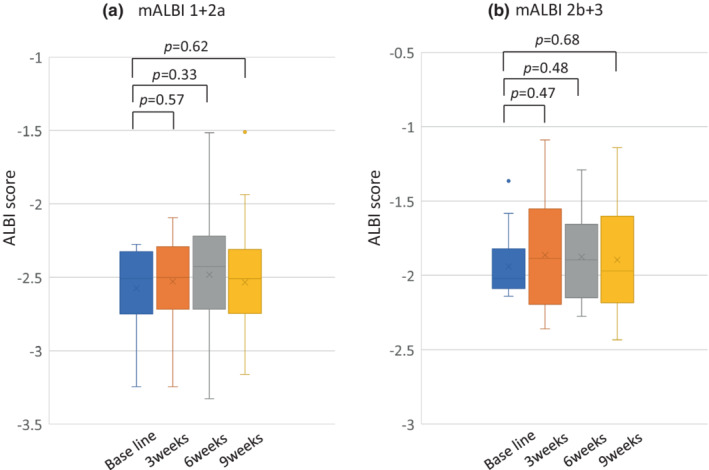
(A) The transition of hepatic functional reserve in mALBI 1+2a after the introduction of treatment with atezolizumab+bevacizumab at baseline, 3, 6, and 9 weeks. (B) The transition of hepatic functional reserve in mALBI 2b+3 after the introduction of treatment with atezolizumab+bevacizumab at baseline, 3, 6, and 9 weeks.

After initiation of Atezo+Bev, the changes in ALBI score were evaluated in 46 patients who were able to continue treatment through week 9 without discontinuation or withdrawal. The median ALBI scores at baseline, 3, 6, and 9 weeks were −2.31 (Quartile, −2.07 to −2.57), −2.31 (Quartile, −2.11 to −2.57), −2.22 (Quartile, −1.98 to −2.62), and −2.36 (Quartile, −1.99 to −2. 62). ALBI scores were not significantly different from baseline at weeks 3, 6, and 9, respectively (Figure 3). When these patients were analyzed in two groups, the mALBI 1+2a group (Figure 4A) and the mALBI 2b+3 group (Figure 4B), there was no significant difference in ALBI score from baseline to week 9 in both mALBI 1+2a and mALBI 2b+3 groups.

### 3.7 Univariate and multivariate analysis of clinical factors affecting the prognosis of Atezo+Bev treatment

Univariate analysis of baseline clinical characteristics identified that the etiology, mALBI grade, and AFP level were contributing factors for better PFS in u‐HCC patients treated with Atezo+Bev (*p* = 0.07, *p* = 0.0058, and *p* = 0.06, respectively) (Table 6). In multivariate analysis, mALBI grade (HR 2.086, *p* = 0.035) was found to be a significant independent factor for PFS in u‐HCC patients treated with Atezo+Bev (Table 6).

**TABLE 6:** Univariate and Multivariate analysis of factors affecting progression‐free survival

Factors	Category	No. of patients	Median PFS (days)	Univariate	Multivariate	
				*p*‐value	Hazard ratio (95% confidence interval)	*p*‐value
Age (years)	≥75	31	144	0.77		
	<75	40	259			
Sex	Male	58	126	0.16		
	Female	13	NA			
Etiology	Viral	37	293	0.07	0.748 (0.376–1.487)	0.41
	No‐viral	34	91			
ECOG‐PS	1	29	112	0.37		
	0	42	259			
mALBI Grade	≥2b	31	91	0.0058	2.086 (1.054–4.130)	0.035
	1, 2a	40	320			
Portal vein invasion	Yes	15	96	0.47		
	No	56	214			
Extrahepatic spread	Yes	24	88	0.55		
	No	47	187			
AFP level (ng/ml)	≥400	25	96	0.06	1.599 (0.833–3.069)	0.16
	<400	46	259			
BCLC C	Yes	37	88	0.11		
	No	34	259			
Treatment line	First line	34	NA	0.41		
	Late line	37	133			

Abbreviations: AFP, alpha‐fetoprotein; BCLC, Barcelona Clinic Liver Cancer; ECOG‐PS, Eastern Cooperative Oncology Group performance status; mALBI, modified albumin–bilirubin; NA, not applicable; PSF, progression free survival.

## 4 DISCUSSION

In this study, Atezo+Bev treatment showed significantly better ORR and PFS in patients with mALBI grade 1 or 2a than those with mALBI grade 2b or 3. Multivariate analysis revealed that mALBI 1+2a was an independent prognostic factor. Additionally, the transition rate to MTAs as secondary treatment was higher in the mALBI 1+2a group than in the mALBI 2b+3 group. These results suggest that starting Atezo+Bev with a good hepatic functional reserve could be more effective for sequential treatment. This study is the first to report an association between the antitumor effects of Atezo+Bev and mALBI.

The anti‐tumor efficacy of Atezo+Bev by RECIST was reported to be 27.3% ORR and 73.6% DCR in the IMbrave150 study,^3^ both of which are generally consistent with our data. There have been several reports on the relationship between background factors and anti‐tumor effects in Atezo+Bev patients in the first and later lines, and many of them reported that there was no significant difference between them.^25^, ^26^ However, when we divided our patients into the mALBI Grade 1+2a and 2b+3 groups for analysis, the mALBI Grade 1+2a group showed statistically significantly better results in both ORR and PFS (Table 2; Figure 2). Although the relationship between the hepatic functional reserve and anti‐tumor efficacy of MTAs for unresectable advanced liver cancer has been reported in several studies,^27^, ^28^, ^29^, ^30^ no reports have analyzed the relationship between mALBI grade and anti‐tumor efficacy in Atezo+Bev therapy. A possible explanation for these results is that there were many cases of withdrawal and discontinuation in our study due to AEs in the mALBI Grade 2b+3 group, which may have affected the anti‐tumor effect. Furthermore, the mALBI 1+2a group was administered more frequently and for longer periods than the mALBI 2b+3 group. This tendency has been shown for MTAs as well, and patients with low hepatic functional reserve reportedly tend to have more AEs, difficulty in dose maintenance, and decreased anti‐tumor efficacy.^28^, ^30^

Because the mALBI score is calculated based on total bilirubin and albumin levels, the mALBI‐2b+3 group tends to include cases with low serum albumin levels. It is possible that these cases tended to show decreased nutritional and performance status, which may have contributed to the increased fatigue and anorexia.^31^, ^32^, ^33^

Recently, the importance of sequential therapy as a therapeutic strategy in pharmacotherapy for HCC has been reported.^34^, ^35^ In addition, the transition rate from LEN to MTAs has been reported to be 43.8% in actual clinical practice with CP‐A and PS‐0,1 as the transition conditions.^36^ The transition rate from Atezo+Bev to systemic chemotherapy was reported by Yoo et al. to be 77.8%; furthermore, Hayakawa et al. reported an 88.2% transition rate to antitumor therapy including TACE and HAIC. However, there have been no reports on treatment transition rates in MTA‐treatable CP‐A and PS‐0,1 patients.^37^, ^38^ In the current study of 41 patients presenting with PD, 46.3% (*n* = 19) were indicated for MTA treatment after progression with Atezo+Bev therapy. However, patients who started treatment with mALBI Grade 1+2a had a non‐significant decrease in mALBI score at the time of PD determination on imaging, and the transition rate to MTA was as high as 60%, suggesting the effectiveness of starting treatment with good liver functional reserve. The mALBI 2b patients who were able to receive Atezo+Bev for nine consecutive weeks had no deterioration of hepatic reserve function (Figure 4B), but those who had imaging PD during treatment showed a trend toward decreased hepatic reserve function. This was thought to be due to the fact that the patients had a background of decreased hepatic reserve; in addition, the exacerbation of tumor factors further reduced the hepatic reserve. Treatment after Atezo+Bev is currently an unmet need though, the effectiveness of MTA therapy after ICI remains unclear. Therefore, a large‐scale analysis, including clarifying the condition of transition to later MTA therapy, is needed in the future.^38^, ^39^

Hepatic functional reserve reportedly tends to decrease once and then improve again during Atezo+Bev treatment.^25^, ^37^ The same trend was observed in the present analysis, with a downward trend at 3 and 6 weeks but an improving trend in the liver reserve at 9 weeks. One possible reason for the primary decrease in the mALBI score may be that the potent anti‐VEGF effect of bevacizumab causes tumor ischemia and inflammation; inflammation is likely to cause injury to the vascular endothelium and hypoalbuminemia as an expression of the inflammatory process.^40^, ^41^ The temporary worsening of the hepatic functional reserve may be followed by an improving trend after the changes associated with the initial anti‐tumor effect have subsided.

Therefore, Atezo+Bev tends to maintain the hepatic functional reserve with a more negligible effect of medication on the hepatic functional reserve, and it is easier to move on to second‐line treatment with MTAs when started in patients with better hepatic functional reserve.

The limitations of our study include its small sample size, short observation period, and retrospective nature. Therefore, it is necessary to examine the findings of this study through a large‐scale prospective study.

Our data suggest that Atezo+Bev treatment has a favorable antitumor effect on mALBI 1+2a, with a relatively good transition rate to subsequent MTA treatment. This would also suggest that it is important to initiate drug therapy while the patient has relatively good liver function reserve to improve the prognosis of patients in the current treatment strategy for u‐HCC.

## AUTHOR CONTRIBUTIONS

Tetsu Tomonari, study concept and design, acquisition of data, statistical analysis, and drafting of the manuscript. Joji Tani, acquisition of data. Yasushi Sato statistical analysis and revision of manuscript. Hironori Tanaka acquisition of data. Takahiro Tanaka acquisition of data. Tatsuya Taniguchi acquisition of data. Asahiroi Morishita, acquisition of data. Koichi Okamoto acquisition of data. Masahiro Sogabe, acquisition of data. Hiroshi Miyamoto, acquisition of data. Naoki Muguruma, acquisition of data. Tsutomu Masaki, acquisition of data. Tetsuji Takayama, revision of manuscript. All authors had access to the data and participated in the writing of this manuscript.

## CONFLICT OF INTEREST

The authors declare no competing interest.

## ETHICS APPROVAL

This study was approved by the Institutional Ethics Committee of the University of Tokushima Hospital (approval number: 3816) and the participating institution.

## INFORMED CONSENT

Informed consent was obtained in the form of opt‐out on the website.

## Supporting information


Figure S1

Figure S2

## Data Availability

The data that support the findings of this study are available from the corresponding author upon reasonable request.
