# Supplementary figures and images for: Initial therapeutic results of atezolizumab plus bevacizumab for unresectable advanced hepatocellular carcinoma and the importance of hepatic functional reserve
Source: Cancer Med. 2022 Aug 14;12(3):2646–57. doi: 10.1002/cam4.5145 (PMC9939118; doi:10.1002/cam4.5145)

Supplementary Figure.1

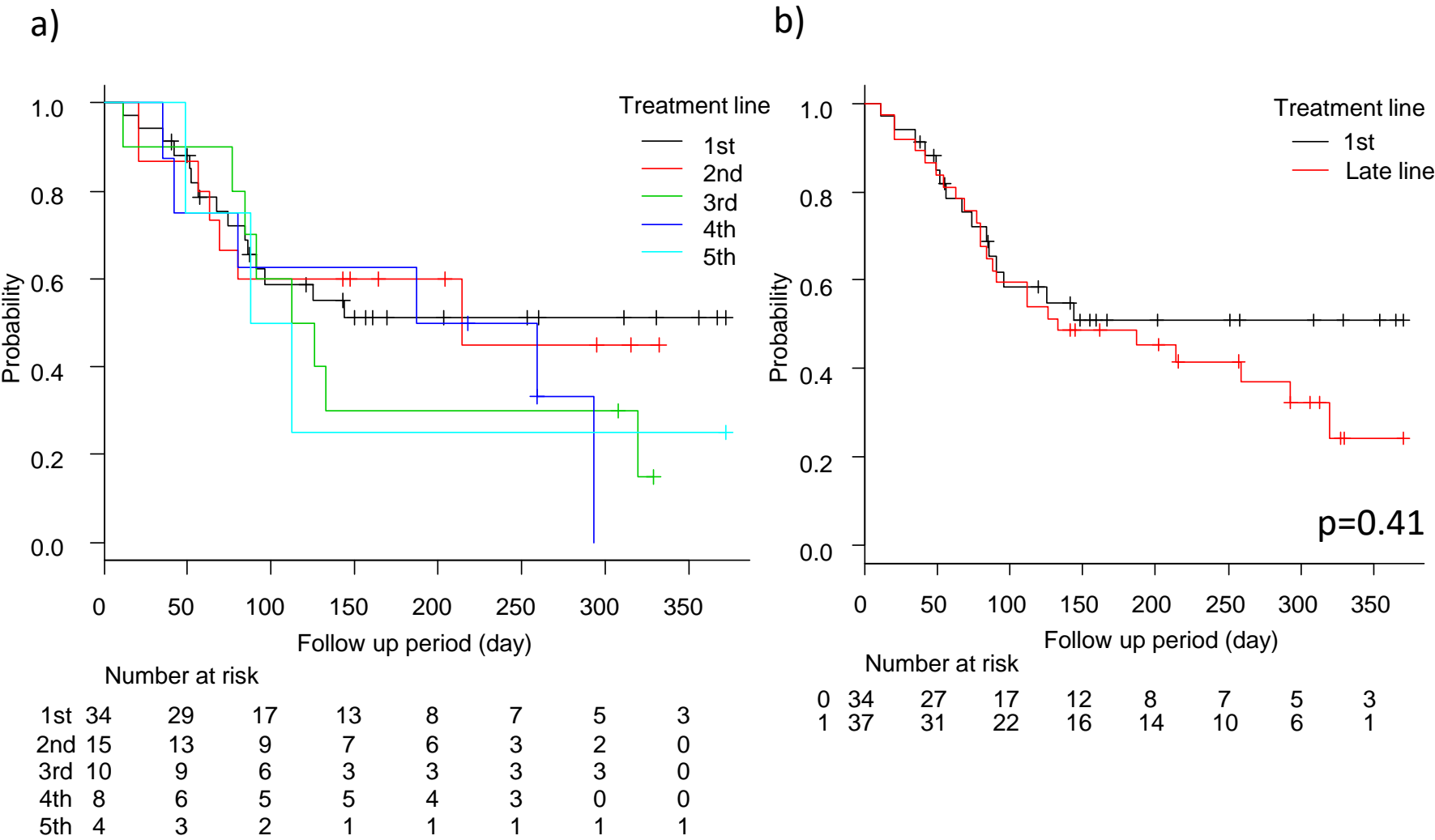

Supplement: Supplementary file 1 — Figure S1 [file CAM4-12-2646-s001.pdf]

Supplementary Figure.2

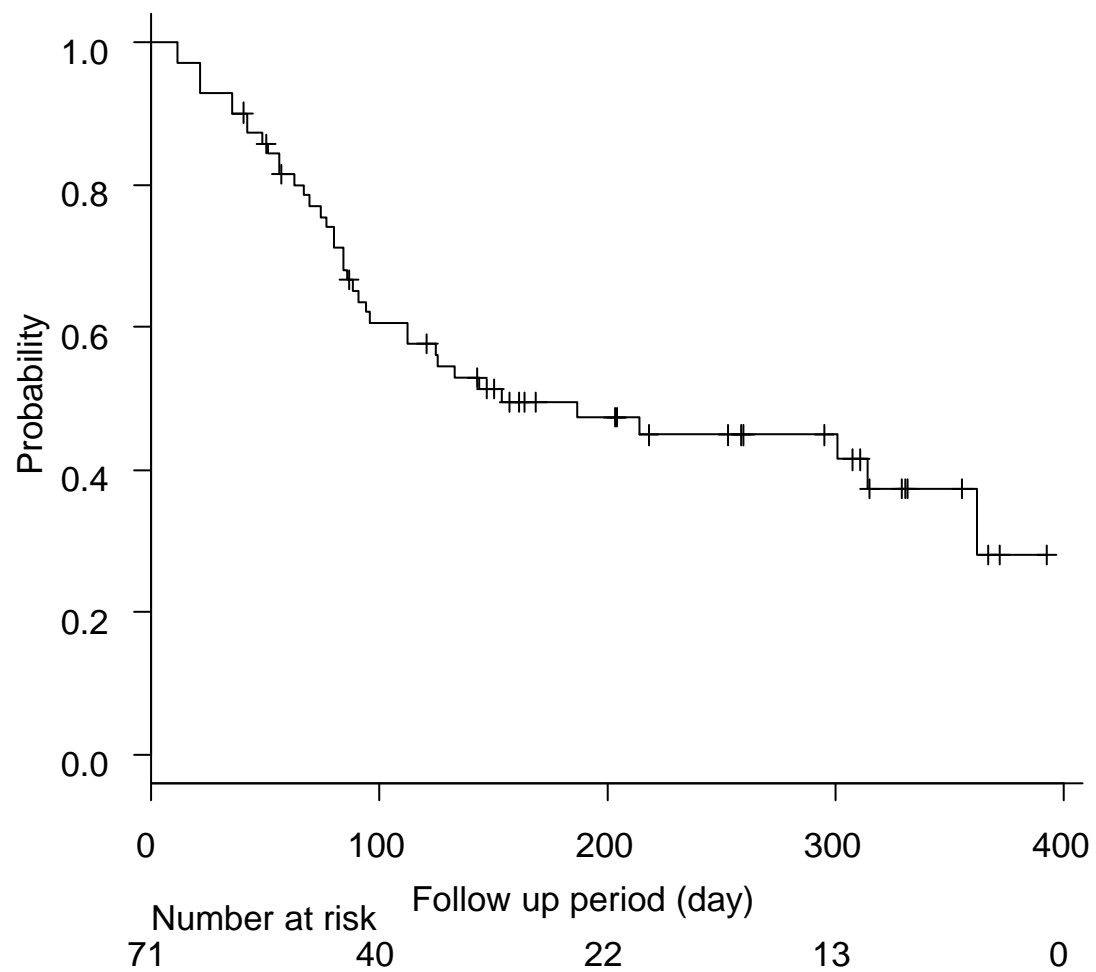

Supplement: Supplementary file 2 — Figure S2 [file CAM4-12-2646-s002.pdf]
